# Evaluating and Improving Surgical Site Infection Prevention: Outcomes of a Two-Cycle Audit and Targeted Interventions in a Low-Resource Setting

**DOI:** 10.7759/cureus.75962

**Published:** 2024-12-18

**Authors:** Basim J Busada

**Affiliations:** 1 General Surgery, Kasr El Ainy, Cairo University, Cairo, EGY

**Keywords:** antibiotics, audit, developing countries, guideline compliance, low income countries, medical education, ssi, ssi prevention, who guidelines

## Abstract

Background

Surgical site infections (SSIs) are among the most common healthcare-associated infections (HAIs), leading to significant morbidity, mortality, and increased healthcare costs. Despite the existence of international guidelines, adherence to best practices remains inconsistent, particularly in low- and middle-income countries (LMICs).

Objective

The objectives of this study are to evaluate compliance with SSI prevention guidelines among medical professionals at Kasr El Ainy Teaching Hospital in Cairo, Egypt, identify gaps in practice and knowledge, and implement targeted interventions to improve outcomes.

Methods

A prospective two-cycle audit was conducted over a period of eight months involving 225 elective surgical patients and 229 healthcare providers. Questionnaires were distributed to foundation year (FY) doctors and surgical residents to assess their knowledge and practices related to SSI prevention across the preoperative, intraoperative, and postoperative phases. Patients were followed up to monitor the incidence of SSIs. Interventions implemented included educational sessions, dissemination of posters, and hands-on training to enhance adherence to best practices.

Results

The initial audit revealed an SSI rate of 12.6% and significant gaps in guidelines knowledge, including improper preoperative hair removal methods and prolonged postoperative antibiotic use (85.7%). Post-intervention, SSI rates decreased to 6.6%, with the questionnaire results showing marked improvements in understanding of key components in SSI prevention: 100% became aware of appropriate intraoperative surgical antibiotic prophylaxis (SAP) (compared to 72.3%), a significant increase (p < 0.05) in the use of clippers (rather than razors) for hair removal (72.1% vs. 33.7%) and intraoperative (rather than preoperative) hair removal (91.8% vs. 10%) (p < 0.05), as well as the need for intraoperative warming practices (87.7% vs. 47.7%) (p < 0.05). Knowledge and adherence to aseptic techniques improved across all staff groups, with awareness increasing from 36.3% to 81.9% among junior doctors, representing a significant improvement (p < 0.05).

Conclusion

Targeted interventions, including education and practice standardization, significantly reduced SSI rates and improved compliance with prevention guidelines. These findings highlight the value of structured audits and educational programs in enhancing surgical outcomes and reducing the burden of SSI, particularly in resource-limited settings.

## Introduction

Surgical site infections (SSIs) are a type of healthcare-associated infection (HAI), which refer to infections that occur as a result of healthcare delivery and can significantly impact patient safety and quality of life. SSIs include any infection that occurs postoperatively at the surgical site within 30 days after surgery or 90 days if an implant is involved [1]. These infections not only result in significant patient morbidity and mortality (M&M) but also have a greater impact on the healthcare system by increasing costs and lengthening hospital stays [2,3]. According to WHO, SSIs are the leading HAI reported in low- and middle-income countries (LMICs) [2]. In comparison, they were reported to be the second and third most prevalent HAIs in the United States and Europe, respectively [1]. For every 100 hospitalized patients at a given time, 22% are expected to develop an SSI, with over 65% of these cases occurring in developing countries [2]. Given these high rates in low-resource settings, studying and addressing this issue to improve outcomes is of great significance.

Despite its importance, comprehensive guidelines on SSI prevention were not published until 2016, when the WHO released its first global guidelines [2]. Other regions followed suit, with the United States introducing the Centers for Disease Control and Prevention (CDC) guidelines for prevention of SSI in 2017 and the UK releasing the National Institute for Health and Care Excellence (NICE) guidelines on SSI prevention and treatment in 2019 [4,5]. 

These guidelines subdivide SSI prevention into three key phases: preoperative, intraoperative, and postoperative [2,5]. Adherence to these guidelines should prevent the occurrence of SSI and improve outcomes. This audit aimed to identify weaknesses and inconsistencies in current practices at Kasr El Ainy Teaching Hospital and implement improvements where significant inconsistencies were identified in wound care knowledge and practice, particularly among junior medical staff. To address these gaps, a two-cycle audit was conducted to evaluate current practices against established guidelines, implement targeted interventions, and assess their effectiveness.

## Materials and methods

Study design 

This prospective snapshot audit was conducted at Kasr El Ainy Teaching Hospital with two primary objectives: (1) to evaluate current SSI prevention practices and the incidence of SSIs among patients undergoing elective procedures and (2) to assess the knowledge and understanding of SSI prevention among medical professionals, including foundation year (FY) doctors and junior and senior surgical residents. SSI detection was based on the WHO classification, which categorizes infections into superficial, deep, and organ-space SSIs according to the depth and tissues involved. Key signs of an SSI include purulent drainage, localized pain or tenderness, swelling, redness, warmth, wound dehiscence, and systemic symptoms such as fever, chills, or tachycardia [2]. A questionnaire, aligned with WHO guidelines on SSI prevention (Table 1) [2], was designed to evaluate knowledge among participants.

**Table 1:** Summary of 2018 WHO guidelines for the prevention of surgical site infection Level of evidence (LoE) and recommendation strength is based on the following: - High LoE: Based on multiple high-quality studies or meta-analyses. - Moderate LoE: Supported by moderate-quality studies or some limitations in study design. - Low LoE: Limited evidence or significant variability in study quality. - Strong recommendation: Benefits clearly outweigh risks; applicable in most settings. - Conditional recommendation: Context-specific; requires consideration of resources and patient factors. Global Guidelines for the Prevention of Surgical Site Infection. Geneva: World Health Organization; 2018.

Phase	Prevention measure	Recommendation	LoE	Strength
Preoperative	Patient preparation	Encourage preoperative bathing or showering with soap or antiseptic	Moderate	Conditional
	Hair removal	Avoid hair removal unless necessary; use clippers, not razors	Moderate	Strong
	Antibiotic prophylaxis	Administer within 1 hour before incision, tailored to procedure and pathogens	High	Strong
	Glucose control	Optimize glycemic control, particularly in diabetic patients	Low	Conditional
	Smoking cessation	Encourage cessation at least 4 weeks before surgery	Moderate	Conditional
	Skin antisepsis	Use alcohol-based antiseptic agents (e.g., chlorhexidine-alcohol)	High	Strong
	Bowel preparation	For colorectal surgery, use combined mechanical bowel prep and oral antibiotics	High	Strong
Intraoperative	Aseptic technique	Maintain strict asepsis, including sterile drapes and proper hand hygiene	Moderate	Strong
	Intraoperative oxygen use	Deliver 80% FiO2 intraoperatively and for 2-6 hours postoperatively (general anesthesia cases)	Moderate	Conditional
	Normothermia	Maintain normal body temperature during surgery	High	Strong
	Drapes and gowns	Use sterile disposable or reusable drapes and gowns	Moderate	Strong
	Use of surgical gloves	Ensure proper glove usage; change if contamination is suspected	Low	Conditional
	Incisional wound irrigation	Consider povidone-iodine irrigation before closure (clean/clean-contaminated cases)	Moderate	Conditional
	Surgical technique	Minimize tissue trauma and ensure effective hemostasis	High	Strong
Postoperative	Wound dressing	Cover wounds with sterile dressing for 48 hours postoperatively	Moderate	Strong
	Drain management	Remove drains as soon as clinically indicated, typically within 24-48 hours	Low	Conditional
	Antibiotics	Avoid routine postoperative antibiotics for clean/clean-contaminated procedures	High	Strong
	Monitoring and education	Educate patients on wound care and infection signs	Low	Conditional

The study aimed to compare current clinical practices and professional knowledge with WHO recommendations, identify gaps or areas for improvement, and propose targeted interventions to enhance adherence and patient outcomes.

Sample size and data collected 

This audit involved a total of 225 patients undergoing surgical procedures, selected based on the following criteria:

Inclusion Criteria

(1) Patients undergoing elective general surgical procedures.

(2) Clean or clean-contaminated procedures (Table 2).

Exclusion Criteria

(1) Patients undergoing emergency general surgery procedures.

(2) Contaminated or dirty procedures (Table 2).

(3) Immunocompromised patients.

(4) Patients with underlying infections.

**Table 2:** Wound classification Wound classification into four classes as per the Centers for Disease Control and Prevention (CDC) [6].

Type of surgery	Definition	Examples	Infection risk
Clean	No inflammation, non-infected surgical site, and no entry into respiratory, GI, GU, or oropharyngeal tracts	Hernia repair and mastectomy	1-5%
Clean-contaminated	Entry into the respiratory, GI, GU, or oropharyngeal tracts under controlled conditions without significant contamination	Cholecystectomy and elective bowel resection	3-11%
Contaminated	Open, fresh accidental wounds; operations with major breaks in sterile technique; or spillage from the GI tract	Penetrating abdominal trauma and bowel perforation	10-17%
Dirty/infected	Existing infection or perforated viscera; surgery involves necrotic tissue or pus	Abscess drainage and infected gangrenous bowel	>27%

The questionnaire included 229 medical professionals, including FY doctors and junior and senior surgical registrars. The sampling method used was simple random sampling, ensuring a representative and balanced selection of participants from both cycles. The questionnaire was split into four subdivisions, each targeting a specific component of SSI prevention:

(1) General information and knowledge about SSI: This included questions about the respondent’s background, basic understanding of SSI, wound types, and confidence in performing wound dressing.

(2) Preoperative care: This included questions about measures to reduce SSI, including hair removal methods, timing and administration of surgical antibiotic prophylaxis (SAP), and indications for suspending immunosuppressive drugs. 

(3) Intraoperative care: This included questions about skin preparation, use of antimicrobial sealants, best practice for scrubbing in, method of skin incision, wound irrigation before closure, routine use of oxygen and warming in decreasing SSI, using sterile vs. reusable gowns, and intraoperative glove changes.

(4) Postoperative care: This included aseptic techniques for wound dressing, prolonged use of SAP, timing of the first dressing, substances for wound dressing, and timing of stitch and drain removal.

Data collection and analysis 

**Figure 1:**
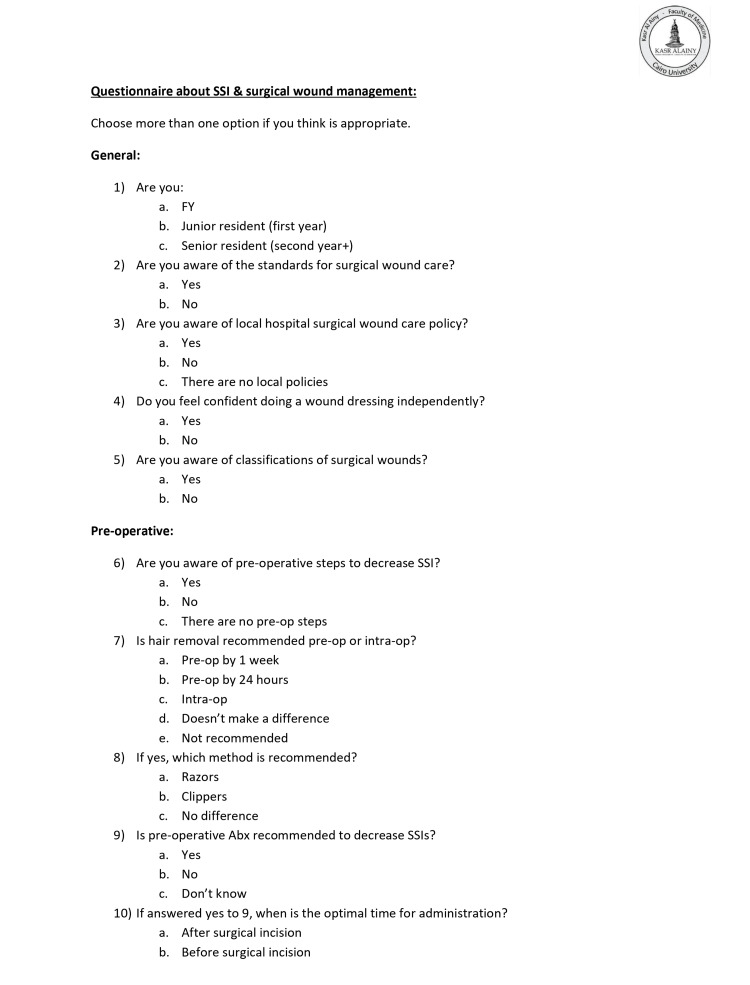
Questionnaire used to assess surgical site infection (SSI) knowledge among doctors Page 1 of the questionnaire

**Figure 2:**
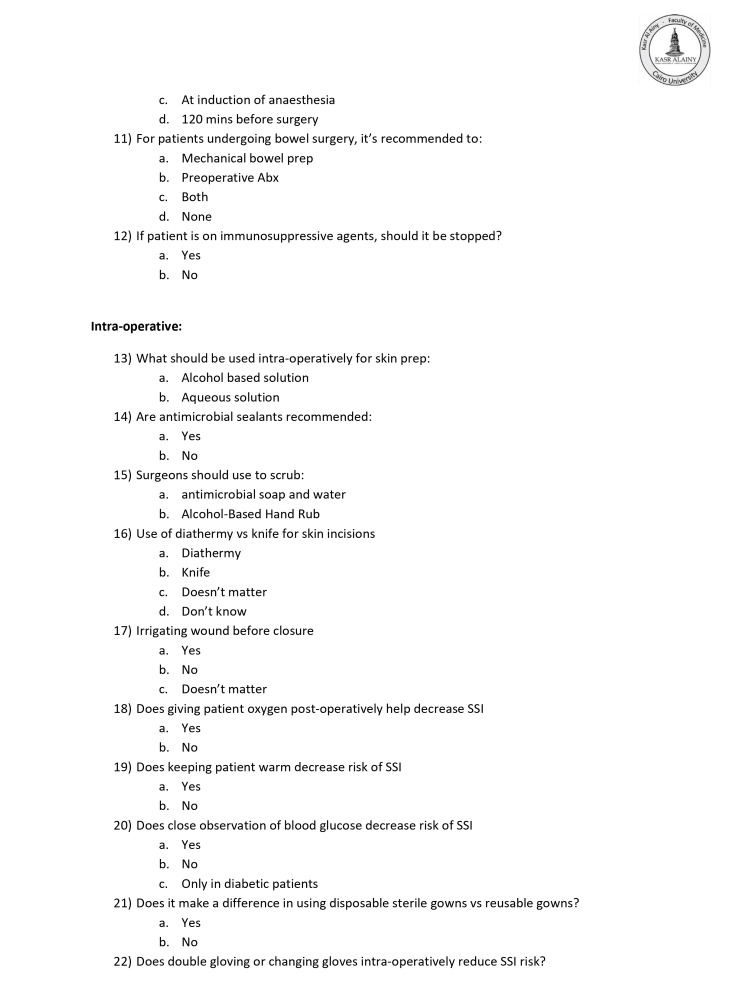
Questionnaire used to assess surgical site infection (SSI) knowledge among doctors Page 2 of the questionnaire

**Figure 3:**
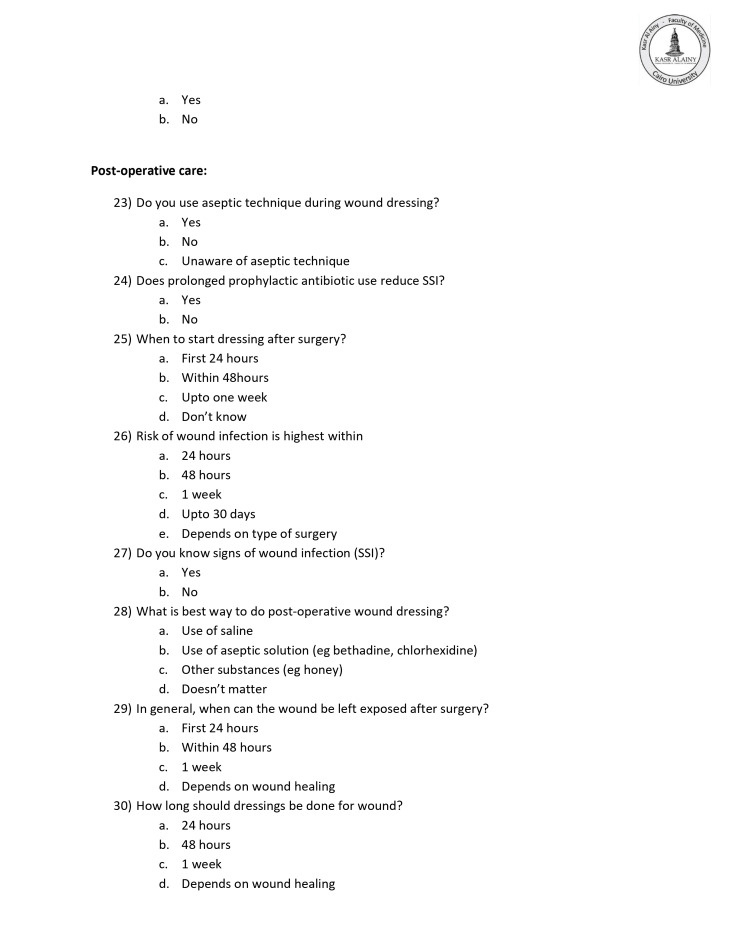
Questionnaire used to assess surgical site infection (SSI) knowledge among doctors Page 3 of the questionnaire

Data were collected using a validated, pre-designed questionnaire to ensure consistency across both audit cycles. The questionnaire, based on WHO guidelines for SSI prevention, consisted of multiple-choice questions divided into four sections: general knowledge, preoperative, intraoperative, and postoperative care. The complete questionnaire is provided in the Appendices (Figures 1-3) for reference. Prior to distribution, the questionnaire was meticulously reviewed to ensure completeness, clarity, and reliability. 

Data were collected by the author and entered into Microsoft Excel, with patient-identifiable information anonymized to maintain confidentiality. All entries were re-verified for accuracy. Basic statistical analyses and graphical representations were conducted in Excel to visualize the findings. A two-proportion z-test was performed to compare the proportions of practices between the first and second audits. A significance level of 0.05 was set, with p-values less than 0.05 considered statistically significant. Proportions were calculated based on the number of participants adhering to each practice, with missing data excluded from the analysis. All statistical analyses were performed using R software [7], and p-values were derived using the normal approximation method for the z-test.

Audit cycles 

The audit was conducted in the following three distinct phases: 

First Cycle

Conducted over a period of three months, this phase involved distributing questionnaires to 107 medical professionals, including 77 FY doctors and 30 junior and senior surgical residents. Data were also collected from 172 elective surgical cases, of which 53 were excluded due to not meeting the inclusion criteria, leaving a total of 119 patients.

Intervention Period

Over the subsequent two months, targeted interventions were implemented to address identified gaps in knowledge and practice:

- Dissemination of audit findings during M&M sessions and journal club meetings. 

- Educational and hands-on sessions focused on aseptic techniques and wound management protocols. 

- Displaying posters in operating theatres, wards, and other communal areas summarizing surgical wound care guidelines.

Second Cycle

Conducted over another three months, this phase mirrored the methodology of the first cycle. Questionnaires were distributed to 122 medical professionals, including 92 FY doctors and 30 surgical residents. Data were collected from 106 patients after excluding 21 who did not meet the inclusion criteria.

Ethical considerations

This audit received formal approval from Cairo University Hospitals Institutional Review Committee (where Kasr El Ainy is an affiliate hospital) under audit number 811/10-23/KSR/CU. All participants provided consent to participate.

## Results

Questionnaire results 

Basic Understanding of SSI Concepts

While surgical residents consistently demonstrated higher levels of understanding of SSI prevention and wound care concepts, the audit revealed significant improvement among FY doctors between the two audit cycles. Notably, knowledge of wound care and SSI among FY doctors improved markedly, rising from 36.3% to 84.7%. Confidence in performing wound dressing also increased substantially, from 66.2% to 85.8%. Awareness of preoperative steps to prevent SSI showed a remarkable improvement as well, with FY doctors advancing from 42.8% to 81.5% and residents peaking at 100%. Finally, there was a marginal increase in knowledge of the local hospital policies on SSI prevention among both groups (Table 3).

**Table 3:** Basic knowledge about SSI among FY and surgical residents This table demonstrates the difference in knowledge about various surgical site infection (SSI) concepts between foundation year (FY) doctors and residents in the first and second audits (post-intervention) based on their responses. The z-values are calculated using a two-proportion z-test. The p-values <0.05 indicate statistically significant differences. --: not applicable

Questions	FY (1st audit)	FY (2nd audit)	z-value	p-value	Residents (1st audit)	Residents (2nd audit)	z-value	p-value
Knowledge of wound care and SSI	36.30%	84.70%	-6.48	<0.05	100.00%	100.00%	--	--
Confidence doing a wound dressing	66.20%	85.80%	-3.01	<0.05	100.00%	100.00%	--	--
Knowledge of classifications of surgical wounds	67.50%	89.10%	-3.45	<0.05	100.00%	100.00%	--	--
Awareness of pre-operative steps to decrease SSI	42.80%	81.50%	-5.22	<0.05	80.00%	100.00%	-2.58	<0.05
Knowledge of local hospital surgical wound care policy	44.00%	50.00%	-0.78	0.437	90.00%	100.00%	-1.78	0.0756

Analysis of the p-value for basic knowledge of SSI revealed a significant improvement (p < 0.05) in all areas for FY doctors, except for knowledge of the local hospital surgical wound care policy, which showed no significant change. For residents, a notable improvement (p < 0.05) was observed in confidence regarding pre-operative steps to reduce SSI (Table 3).

Preoperative and Intraoperative Factors

Similarly, there has been an overall improvement in understanding both preoperative and intraoperative factors in the prevention of SSI post-audit. Overall understanding of removal of hair intra-operatively rather than pre-operatively increased dramatically from 10% to 91.8%, with 72.1% (in comparison to 33.7% pre-audit) advocating the use of clippers rather than shaving. There has also been increased awareness of the limited value of stopping immunosuppressive agents pre-operatively and the use of anti-microbial sealants intra-operatively. Additionally, understanding of the role of maintaining normothermia and providing supplemental oxygen intraoperatively to reduce SSI improved markedly, with awareness rising from 47.6% to 87.7% and 25.2% to 63.9%, respectively (Table 4).

**Table 4:** Knowledge about preoperative and intraoperative factors This table demonstrates the difference in knowledge regarding preoperative and intraoperative factors among doctors between the first and second audits (post-intervention) based on their responses. The z-values are calculated using a two-proportion z-test. The p-values <0.05 indicate statistically significant differences. --: not applicable

Question	First audit	Second audit	z-value	p-value
Hair removal (intra-op)	10.00%	91.80%	-12.34	<0.05
Clippers use	33.70%	72.10%	-5.83	<0.05
Pre-operative antibiotics use	95.30%	91.80%	1.08	0.28
Bowel preparation	100.00%	100.00%	--	--
Stop immunosuppressive agents	77.00%	45.10%	4.86	<0.05
Antimicrobial sealants recommended	73.83%	63.93%	1.61	0.11
Irrigating wound before closure	78.50%	87.70%	-1.87	0.06
Giving patient oxygen	25.23%	63.93%	-5.86	<0.05
Keeping patient warm	47.66%	87.70%	-6.54	<0.05
Close observation of blood glucose	70.09%	89.34%	-3.66	<0.05
Using disposable sterile gowns vs. reusable gowns	40.19%	18.85%	3.56	<0.05
Double gloving or changing gloves intra-operatively	14.95%	25.41%	-1.95	0.05

Analysis of preoperative and intraoperative knowledge revealed significant differences in all factors except for antimicrobial sealants, wound irrigation before closure, and glove changing intra-operatively which still represented gaps in knowledge among doctors (Table 4).

*Postoperative Factors* The audit highlighted significant improvements in addressing misconceptions regarding postoperative wound management among both junior and senior doctors. Key areas of progress included a better understanding of the inappropriate long-term use of prophylactic antibiotics, the optimal timing for the first dressing change, and the appropriate agents for wound dressing. Notably, 57.3% of participants recognized that saline is sufficient for dressing clean wounds, a concept entirely unrecognized before the audit. Moreover, knowledge of aseptic techniques during wound dressing has nearly doubled, increasing from 40% to 80%. However, gaps remain; nearly 50% of respondents still believed that prolonged antibiotic use was recommended, and 42.6% incorrectly suggested exposing the wound within 24 hours post-surgery, underscoring areas for further improvement (Table 5).

**Table 5:** Knowledge about postoperative factors This table demonstrates the difference in knowledge regarding postoperative factors among doctors between the first and second audits (post-intervention) based on their responses. The z-values are calculated using a two-proportion z-test. The p-values <0.05 indicate statistically significant differences.

Question	First audit	Second audit	z-value	p-value
Use of aseptic technique	43.90%	81.90%	-5.99	<0.05
Long-term prophylactic antibiotic use	84.10%	49.10%	5.55	<0.05
Dressing (first 24 hours)	72%	26.22%	6.91	<0.05
Dressing (first 48 hours)	15%	58.20%	-6.73	<0.05
Recognition of signs of SSI	86.90%	87.70%	-0.18	0.86
Use of saline in dressing	0%	57.30%	-9.4	<0.05
Exposing wound (first 24 hours)	87%	42.60%	6.94	<0.05
Exposing wound (first 48 hours)	10.20%	50.80%	-6.57	<0.05
Wound dressing (1 week)	87%	67.20%	3.5	<0.05

The most notable improvement was observed in the understanding of postoperative concepts, with significant gains in all factors except for the recognition of signs of SSI (Table 5). This lack of improvement may be attributed to the initially high level of knowledge on this topic, with over 85% of participants demonstrating awareness.

Patient results

A total of 299 patients were initially included in the study. After excluding 74 patients who did not meet the inclusion criteria, 119 patients were part of the first audit, and 106 patients were included in the second audit. Of the patients in the first audit, 12.6% (15) developed postoperative SSIs. Following the interventions, this number markedly decreased to 6.6% (seven) in the second audit. Additionally, the use of SAP showed remarkable improvement, increasing from 72.3% to full compliance at 100%. Furthermore, there was a substantial reduction in the use of postoperative long-term antibiotics, dropping from 85.7% to 21.7% (Table 6).

**Table 6:** Patient data This table shows outcomes in terms of surgical site infection (SSI) rate, timely use of surgical antibiotic prophylaxis (SAP), and long-term use of antibiotic prophylaxis between the first and second audits (post-intervention). The z-values are calculated using a two-proportion z-test. The p-values <0.05 indicate statistically significant differences.

Variable	First audit	Second audit	z-value	p-value
Proportion of SSIs	12.6%	6.6%	1.5786	0.1144
Timely use of SAP	72.30%	100.00%	-5.8626	<0.005
Long-term use of prophylactic antibiotics	85.70%	21.70%	9.6344	<0.005

Analysis of the data revealed a significant p-value for the timely introduction of SAP and a reduction in the use of extended surgical antibiotics. However, no significant reduction in SSI rates was observed. This might have been limited by the sample size (Table 6).

## Discussion

SSIs represent a major burden on healthcare systems worldwide. According to the WHO, SSI in US hospitals in 2005 resulted in more than 928,000 additional hospital stays [2]. In terms of cost, each SSI case is estimated to cost approximately USD 20,785, accounting for one-third of all HAI expenses in the United States. This makes SSI the most costly HAI, with an estimated annual cost of USD 3.3 billion [8]. 

Being such a massive issue, multiple evidence-based guidelines have been implemented to enable healthcare providers to prevent SSI [4,9,10]. Most of these guidelines share similar recommendations with some minor differences that primarily reflect local findings (Table 7). The main differences primarily lie in their focus on timing, resource sensitivity, and specific interventions. For instance, for prophylactic antibiotics, WHO allows up to 120 minutes before incision, while others recommend stricter administration within 60 minutes. Preoperative bathing also varies, with the CDC favoring chlorhexidine-based solutions, while the WHO and NICE are less prescriptive. Smoking cessation is universally encouraged, but only the WHO, CDC, and American College of Surgeons and Surgical Infection Society (ACS/SIS) specify a four-week cessation period. In the intraoperative phase, all guidelines emphasize normothermia and alcohol-based skin antiseptics, but the NICE is more cautious about antiseptic sutures and wound irrigation, citing limited evidence. Postoperatively, the WHO and NICE recommend sterile dressings for 48 hours, whereas the CDC and ACS/SIS prefer breathable or transparent options. Overall, the differences reflect regional healthcare priorities, with the WHO emphasizing flexibility for resource-limited settings, the NICE tailored to the UK context, and the CDC and ACS/SIS offering more specific, evidence-based recommendations for their respective regions [2,4,5,11].

**Table 7:** Comparison of SSI prevention guidelines from the WHO, NICE, CDC, and ACS/SIS This table summarizes key recommendations from five major guidelines on the prevention of surgical site infections (SSI): the World Health Organization (WHO) guidelines [2], the National Institute for Health and Care Excellence (NICE) guidelines [4], the Centers for Disease Control and Prevention (CDC) guidelines [5], and the American College of Surgeons (ACS) and Surgical Infection Society (SIS) guidelines [11]. It highlights common practices and key differences across the guidelines, focusing on the preoperative, intraoperative, and postoperative phases of SSI prevention.

Phase	Aspect	WHO	NICE	CDC	ACS/SIS
Preoperative	Antibiotics timing	Within 120 minutes	Within 60 minutes.	Within 60 minutes.	Within 60 minutes.
	Bathing	Soap/antiseptic; flexible	Soap unless contaminated	Chlorhexidine preferred	Antiseptic where possible
	Smoking cessation	≥4 weeks pre-op	Encouraged, no timeline	≥4 weeks pre-op	≥4 weeks pre-op
	Hair removal	Clippers if necessary	Clippers if necessary	Clippers if necessary	Clippers only
	Glucose control	Focus on perioperative glycemia	Tight control advised	Similar to WHO and NICE	Perioperative control key
	Skin antiseptics	Alcohol-based (e.g., chlorhex)	Alcohol-based recommended	Alcohol-based preferred	Supports alcohol-based prep
Intraoperative	Normothermia	Maintain active warming	Strongly recommended	Active warming required	Emphasizes warming methods
	Antiseptic sutures	Supports triclosan sutures	Based on evidence	Recommends triclosan sutures	Strongly supports
	Wound irrigation	Supports antiseptics (e.g., iodophor)	Limited evidence	Antiseptics or saline if needed	Antiseptics for high-risk
	Oxygenation	Recommends supplemental O₂	No specific advice	Encourages perioperative O₂	Supports O₂ optimization
	Surgical drapes	Sterile, single-use	Sterile, single-use	Sterile, single-use	Strongly endorsed
Postoperative	Dressings	Sterile for 48 hours	Sterile, non-occlusive	Sterile, breathable	Allows occlusive if needed
	Wound care	Aseptic wound care essential	Regular inspections	Aseptic care emphasized	Prioritizes aseptic care
	Antimicrobial stewardship	Targeted use to avoid resistance	Antibiotic use cautiously	Strong focus on stewardship	Reinforces stewardship

Despite these extensive guidelines and their accessibility, compliance with these has been reported to be poor, especially in LMICs [2,12], which represent 63% of the world’s nations and include more than 75% of the world’s population [13]. It’s estimated that, on average, 11.8% of patients undergoing a surgical procedure in LMIC will develop an SSI, with rates going as high as 19% in certain procedures such as cesarean sections [2]. There are multiple factors that contribute to this increased SSI rates in LMICs. Key factors include limited access to resources like sterile surgical supplies, antibiotics, and antiseptics, as well as inadequate water, sanitation, and hygiene (WASH) infrastructure [2,13,14]. In LMICs, the reuse of single-use medical equipment is common, often accompanied by inadequate reprocessing and sterilization protocols. These protocols are frequently poorly regulated and insufficient, compromising the sterility of medical supplies and increasing the risk of infections [15]. Moreover, healthcare systems are usually overburdened, and there are severe shortages of adequately trained staff, which results in inconsistent adherence to infection prevention and control protocols. Finally, many LMICs lack robust SSI surveillance systems, making it difficult to monitor and address infection trends effectively. These challenges, combined with higher rates of malnutrition and comorbidities among patients, significantly increase the risk of SSIs [2,13]. 

However, the issue with compliance with SSI prevention guidelines and care bundles is not only confined to LMICs but also extends to high-income countries. This occurs despite the implementation of various bundles, such as the Surgical Care Improvement Project (SCIP) in the USA, which aims to streamline this process. This lack of compliance stems from the need for substantial changes across multiple levels, including individual attitudes, cultural practices, and institutional systems. Surgeons, in particular, are often identified as a critical factor in non-compliance. Resistance to adopting standardized checklists and guidelines is frequently linked to ingrained personal habits or professional routines. Some practitioners may perceive these tools as undermining their autonomy or questioning their necessity, leading to inconsistent adherence [12]. 

This study conducted in Kasr El Ainy Teaching Hospital highlights a significant lapse in knowledge among medical professionals, including senior surgical residents, who are expected to be well-versed in SSI prevention guidelines as part of their training. Similar findings were reported by Mohsen et al. in a study also conducted in Egypt, which assessed the knowledge of 400 nurses regarding SSI prevention across preoperative, intraoperative, and postoperative phases [16]. The results revealed widespread gaps in understanding, with an average of nearly 70% of questions answered incorrectly. For example, questions on hair removal, a critical aspect of preoperative care, were answered incorrectly by 78.5% of participants. These results mimic the ones presented in this audit, where initially 90% answered incorrectly that hair needs to be removed pre-operatively, and 66.3% supported the use of razors/shavers. This is contrary to the guidelines, which recommend that hair removal should only occur if absolutely necessary and then only with clippers during surgery. Shaving, especially preoperatively, is strongly discouraged [2]. However, after this audit, the understanding of this guideline improved markedly, with nearly 92% of participants acknowledging that hair should only be removed during surgery, and 72% supported the use of clippers rather than shaving. This underlines the importance of audits and feedback formats, a finding that has been reported by Manivannan et al., who evaluated the Surveillance of SSI, Auditing, and Feedback (SAF) effect on the rate of compliance with an SSI care bundle and measured its effectiveness in reducing the SSI rate [17]. They found that there had been a significant reduction in SSI rates (from 10.0% to 1.3%; p < 0.0001) and increased compliance with SSI protocols (from 92.7% to 99%) by the end of their study [17]. 

This audit also identified a significant issue with prolonged use of prophylactic antibiotics postoperatively, which is not recommended for preventing SSIs [2]. Post-intervention, a 64% reduction in the routine use of postoperative antibiotics was observed, which represented a significant reduction (p < 0.05). This reduction not only contributes to cost savings and reduces the risk of antimicrobial resistance, but it also has environmental benefits [18]. According to Spivak et al., in 2022 alone, over 66 million unnecessary antibiotic prescriptions issued in the United States resulted in 1,887 metric tons of CO2 emissions, equivalent to driving 4.8 million miles or circumnavigating the globe nearly 200 times [19]. 

The observed significant improvements in SSI prevention knowledge among medical professionals, reduction of prolonged antibiotic use, and timely SAP administration, along with better SSI outcomes, in this audit, highlight the critical role of audits and feedback in enhancing compliance with SSI guidelines. However, this remains an area requiring further refinement. A 2021 systematic scoping review on feedback data for reducing SSIs and optimizing antibiotic use in surgery identified potential gaps in the feedback provided to medical professionals regarding SSI prevention. It noted that in 60% of cases, limited feedback was provided only to the heads of surgery departments or delivered separately to surgeons, anesthesiologists, or other prescribers. Only a minority (30%) extended feedback to frontline staff, such as operating room personnel, pharmacists, and nurses, highlighting a missed opportunity to engage all key stakeholders in SSI prevention. Despite these gaps, the review demonstrated that most forms of feedback led to significant reductions in SSI rates, reinforcing the necessity of incorporating structured audits and feedback mechanisms into routine practice [20]. 

A systematic review by Pereira et al. shows that many tools can be utilized when attempting to implement new guidelines and changing ongoing habits in medical staff. From these, the most important and efficient included educational materials, educational meetings, reminders, academic detailing, and audits and feedback [21]. Also, as proven by Manivannan et al., audits are very powerful tools [17]. In this experience, conducting this audit and implementing various tools, including discussions in meetings, distributing posters, conducting education sessions, and hands-on sessions, have resulted in a dramatic 50% reduction in SSI rates in a short period of time. 

Another effective strategy to enhance adherence to SSI prevention practices is the implementation of SSI-reducing care bundles. As mentioned previously, several care bundles have been introduced at national levels, such as the SCIP in the United States and the Department of Health’s High Impact Intervention (DH HII) in England [22,23]. A 2015 systematic review, which included 13 studies and 8,515 patients, demonstrated that surgical care bundles significantly reduce the risk of SSIs compared to standard care (p = 0.0005). The SSI rate in the care bundle group was 7.0% (328/4,649) compared to 15.1% (585/3,866) in the standard care group [24]. Conversely, some studies, including Tanner et al., reported no significant reduction in SSI rates with care bundle implementation [25]. However, this was attributed to the already high compliance with SSI prevention recommendations prior to the intervention, which likely limited the potential for further improvement. In contrast, in LMICs, where compliance with SSI prevention measures is generally lower [2], the introduction of care bundles could potentially have substantial benefits in reducing SSI rates.

Finally, an important factor to consider is that the increase in knowledge and awareness of SSI prevention not only contributes to improved patient outcomes but also leads to shorter hospital stays and reduced healthcare costs. While the cost of an SSI in Egypt averages $72.58 per day [26], the financial burden is much higher in other countries, such as the UK, where the cost can reach £536 (~$683) per day [27]. 

Limitations 

The sample size of 229 medical professionals provides a sufficient starting point for initial insights but limits the broader applicability of the findings. Additionally, the targeted sample excluded other key members of the medical team, such as nurses and consultants, who play a significant role in SSI prevention. Being a single-center study conducted solely at Kasr El Ainy Teaching University Hospital, the results may not fully reflect settings with different resources or training systems. Furthermore, the short intervention period may have restricted participants’ ability to gain in-depth knowledge, potentially affecting the retention and long-term implementation of the skills learned. 

From the patient data, the relatively small sample size of 119 patients in the first audit and 106 in the second audit might limit the statistical power of the analysis, particularly for detecting significant reductions in SSI rates. While improvements in SAP compliance and reductions in long-term antibiotic use were evident, the observed reduction in SSI rates did not reach statistical significance, potentially due to the limited number of observed infections. 

## Conclusions

This audit underscores the significant impact of targeted interventions on reducing SSIs and improving adherence to evidence-based guidelines. Through education, awareness campaigns, and practical training, a 50% reduction in SSI rates was achieved, alongside marked improvements in guideline compliance and enhanced understanding of best practices among healthcare professionals. These outcomes highlight the importance of continuous education, structured audits, and evidence-based interventions in improving patient care, reducing healthcare costs, and minimizing the environmental impact associated with unnecessary antibiotic use. The findings support the need for sustained efforts, including routine teaching and possibly the introduction of SSI-reducing bundles, to integrate these practices into routine clinical workflows. This is particularly important in resource-limited settings, where such interventions can lead to lasting improvements in surgical outcomes.
